# Identification of key microRNAs of plasma extracellular vesicles and their diagnostic and prognostic significance in melanoma

**DOI:** 10.1515/med-2020-0111

**Published:** 2020-05-30

**Authors:** Jiachao Xiong, Yan Xue, Yu Xia, Jiayi Zhao, Yuchong Wang

**Affiliations:** Department of Plastic Surgery, Changhai Hospital, Naval Military Medical University, Shanghai 200433, China; Department of Dermatology, Changhai Hospital, Naval Military Medical University, Shanghai 200433, China; Department of General Practice, Changhai Hospital, Naval Military Medical University, Shanghai 200433, China

**Keywords:** bioinformatics, melanoma, plasma extracellular vesicles, differentially expressed genes

## Abstract

Melanoma is one of the most highly metastatic, aggressive and fatal malignant tumors in skin cancer. This study employs bioinformatics to identify key microRNAs and target genes (TGs) of plasma extracellular vesicles (pEVs) and their diagnostic and prognostic significance in melanoma. The gene expression microarray dataset (GSE100508) was downloaded from the Gene Expression Omnibus database. Differential analysis of miRNAs in pEVs was performed to compare melanoma samples and healthy samples. Then, TGs of the differential miRNAs (DE-miRNAs) in melanoma were selected, and differential genes were analyzed by bioinformatics (including Gene Ontology and Kyoto Encyclopedia of Genes and Genomes pathway enrichment, protein–protein interaction network and prognostic analysis). A total of 55 DE-miRNAs were found, and 3,083 and 1,351 candidate TGs were diagnostically correlated with the top ten upregulated DE-miRNAs and all downregulated DE-miRNAs, respectively. Prognostic analysis results showed that high expression levels of hsa-miR-550a-3p, CDK2 and POLR2A and low expression levels of hsa-miR-150-5p in melanoma patients were associated with significantly reduced overall survival. In conclusion, bioinformatics analysis identified key miRNAs and TGs in pEVs of melanoma, which may represent potential biomarkers for the early diagnosis and treatment of this cancer.

## 1 Introduction

Melanoma is one of the most highly metastatic, aggressive and fatal malignant tumors in skin cancer [1]. Melanoma is often systemic and asymptomatic in patients and has become the tumor with the highest rate of brain metastases in adult solid tumors [2]. Although melanoma is rare, it has been reported that the incidence of skin melanoma is increasing worldwide, which has become a health problem that warrants further study [3,4]. The current treatment of cutaneous melanoma is surgical resection, and other treatments include radiotherapy, chemotherapy, immunotherapy and targeted therapy [3,5]. Unfortunately, the treatment of skin melanoma is not satisfactory at present, and the prognosis of patients after treatment is not good [5]. Therefore, it is crucial to identify more effective molecular markers for the early recognition and treatment of cutaneous melanoma to improve the prognosis of patients.

MicroRNAs (miRNAs) are small noncoding RNAs that are generally 18–25 nt in length and are embedded in independent noncoding RNA or introns that encode proteins. Mature miRNA exerts its biological function by combining with ribosomes, similar to the RNA-induced silencing complex, to form miRNA and recognize target genes (TGs). The target of miRNA is polymorphic; that is, a miRNA can have multiple TGs or multiple miRNAs have the same TG. In addition, the change in expression during the formation of miRNA may have a carcinogenic or antitumor effect on the tumor. Therefore, miRNAs may regulate the occurrence and development of tumors by identifying their specific TGs. In recent years, plasma extracellular vesicles (pEVs) have received extensive attention as a means of communication between cells and organs [6]. A large number of studies have found that pEVs are involved in intercellular communication and various physiological and pathological processes [7,8,9]. Studies have shown that EVs extracted from tumor cells have become one of the key factors in the development of malignant tumors by regulating the involvement of target cells in tumor growth, metastasis and immune response [10,11,12]. In addition, accumulating evidence has indicated that miRNAs, as an important component of EVs, play an important role in the development of human malignancies [12]. Therefore, exploring specific miRNAs in pEVs of melanoma patients may be potential molecular targets for the early diagnosis or treatment of melanoma patients.

To date, few studies have investigated the molecular mechanism of miRNAs in the pEVs of patients with melanoma. Thus, in this study, bioinformatics technology was used to identify differential miRNAs (DE-miRNAs) and TGs related to the diagnosis and prognosis of pEVs in patients with melanoma. Subsequently, Gene Ontology (GO) annotation, Kyoto Encyclopedia of Genes and Genomes (KEGG) pathway enrichment analysis, protein–protein interaction (PPI) network and survival analysis were used to screen out the DE-miRNAs and target the genes most relevant to the diagnosis and prognosis of malignant melanoma, which may become potential targets for the early diagnosis, treatment and prognosis analysis of malignant melanoma patients.

## 2 Materials and methods

### 2.1 Microarray data of pEVs in cases of melanoma

The gene expression microarray dataset (GSE100508) was downloaded from the Gene Expression Omnibus (GEO) database (https://www.ncbi.nlm.nih.gov/geo). The dataset was based on the GPL22079 platform (Agilent-031181 Unrestricted Human miRNA V16.0 Microarray), which included samples from 14 healthy controls, 14 patients with melanoma, 15 patients with a high risk of recurrence and 11 with a low risk of recurrence. The miRNA expression data of pEVs in melanoma patients and healthy controls were downloaded and used for this study.

### 2.2 Differential analysis of miRNA expression in pEVs

The series and platform data were converted and loaded through the R language software and the annotation package. Then, the data were standardized by the limma R package’s array function (http://www.bioconductor.org/) [13]. The differential analysis of miRNAs in pEVs between melanoma samples and healthy samples was performed by using the GEO2R analysis tool. An miRNA with a statistical significance *P*-value of <0.05 and a base-2 logarithm of fold change (log FC) value of greater than ±1 was defined as DE-miRNA. The dataset was visualized with Morpheus online tools (https://software.broadinstitute.org/morpheus/) and OriginPro (version 2016), and the integrated DE-miRNA lists of upregulated and downregulated were saved for subsequent analysis [14].

### 2.3 Selection of TGs of DE-miRNAs in melanoma

miRTarBase (http://mirtarbase.mbc.nctu.edu.tw/php/index.php), an experimentally validated miRNA–target interaction database, was used to screen TGs of DE-miRNAs [15]. This study selected the top ten upregulated miRNAs and all downregulated miRNAs. Subsequently, miRTarBase was used to retrieve and screen for TGs associated with DE-miRNAs in melanoma.

### 2.4 GO annotation and KEGG pathway enrichment analysis of TGs

Protein analysis through evolutionary relationship (PANTHER) systems (http://pantherdb.org/) classifies genes or proteins based on their molecular functions (MFs), biological processes (BPs) and pathways to facilitate high-throughput functional analysis [16]. In this study, the PANTHER classification system was used for GO annotation and KEGG pathway enrichment analysis of the integrated TGs. The GO annotation analysis of TGs involved the BP, cell component (CC) and MF of the genes. A *P*-value of <0.05 was considered to be significant.

### 2.5 Integration analysis of the TG interaction network

The predicted TGs of the top five most upregulated and all downregulated DE-miRNAs were selected, and the PPI networks were analyzed with the STRING database (https://string-db.org/) [17]. Then, Cytoscape (version 3.7.2) was used to visualize the PPI networks. Gene networks can be used to study the relationship between genetic factors of disease, in which highly connected nodes are defined as hub genes. The degree analysis method in the CytoHubba plug-in was used to calculate the grade nodes in the PPI network, and the top 25 hub genes were selected and visualized by Cytoscape [18]. The Catalogue of Somatic Mutations in Cancer (COSMIC) database (https://cancer.sanger.ac.uk/cosmic) was used to identify the genes with the largest mutation and variation in melanoma [19]. Subsequently, the bioinformatics prediction miRNA Data Integration Portal (mirDIP) online database (http://ophid.utoronto.ca/mirDIP/) was used to highlight the relative strength of each COSMIC gene and hub gene with DE-miRNAs. The mirDIP database integrates 30 TG databases and uses algorithms to score miRNA–TG interactions with confidence [20]. The confidence class was divided into four levels: very high, high, medium and low. Based on the level of miRNA–TG interaction, the DE-miRNAs most correlated with melanoma could be screened out.

### 2.6 Prognostic analysis associated with hub genes for patients with melanoma

UALCAN (http://ualcan.path.uab.edu/) and OncoLnc (http://www.oncolnc.org/) are interactive webs that provide in-depth analysis of The Cancer Genome Atlas (TCGA) gene expression data [21]. In this study, the top five DE-miRNAs, the COSMIC genes and the top five hub genes most closely related to DE-miRNAs were selected. Then, survival analysis was performed by UALCAN and OncoLnc to screen out the hub genes most relevant to the prognosis of patients with melanoma. A *P*-value of <0.05 was considered to be significant.

## 3 Results

### 3.1 Identification of DE-miRNAs and TGs in pEVs of melanoma

**Figure 1:**
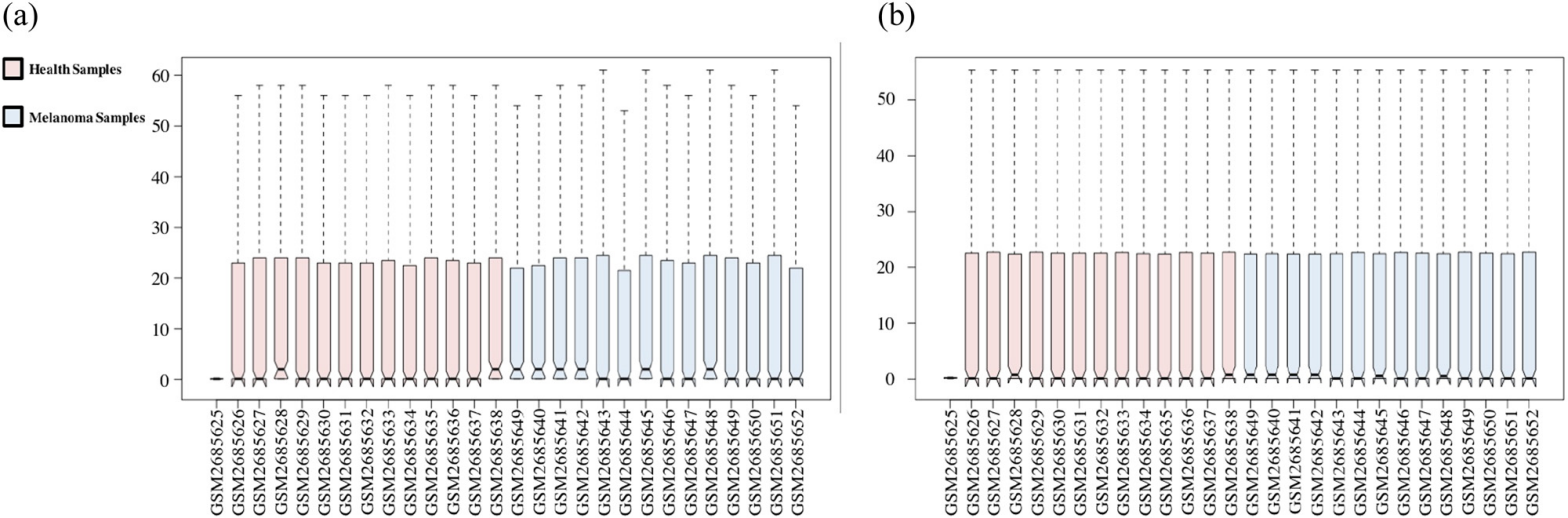
The GSE100508 microarray dataset obtained from the GEO database. (a) The microarray dataset before normalization. (b) The microarray dataset after normalization.

**Figure 2:**
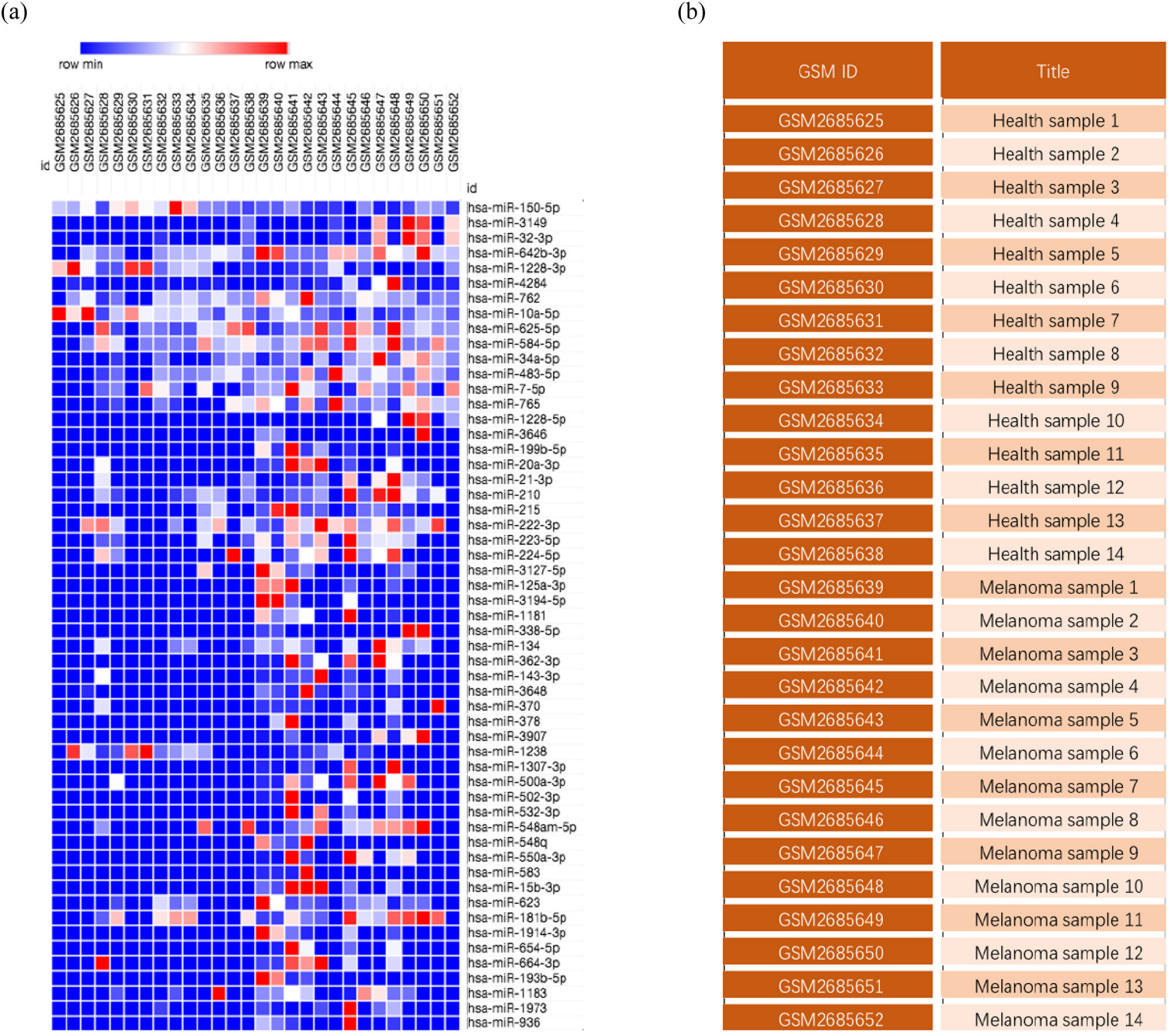
Differential analysis of the miRNA expression in pEVs of melanoma. (a) Heat map of DE-miRNAs in 14 cases of melanoma and 14 health controls. (b) The details of the 28 cases.

**Figure 3:**
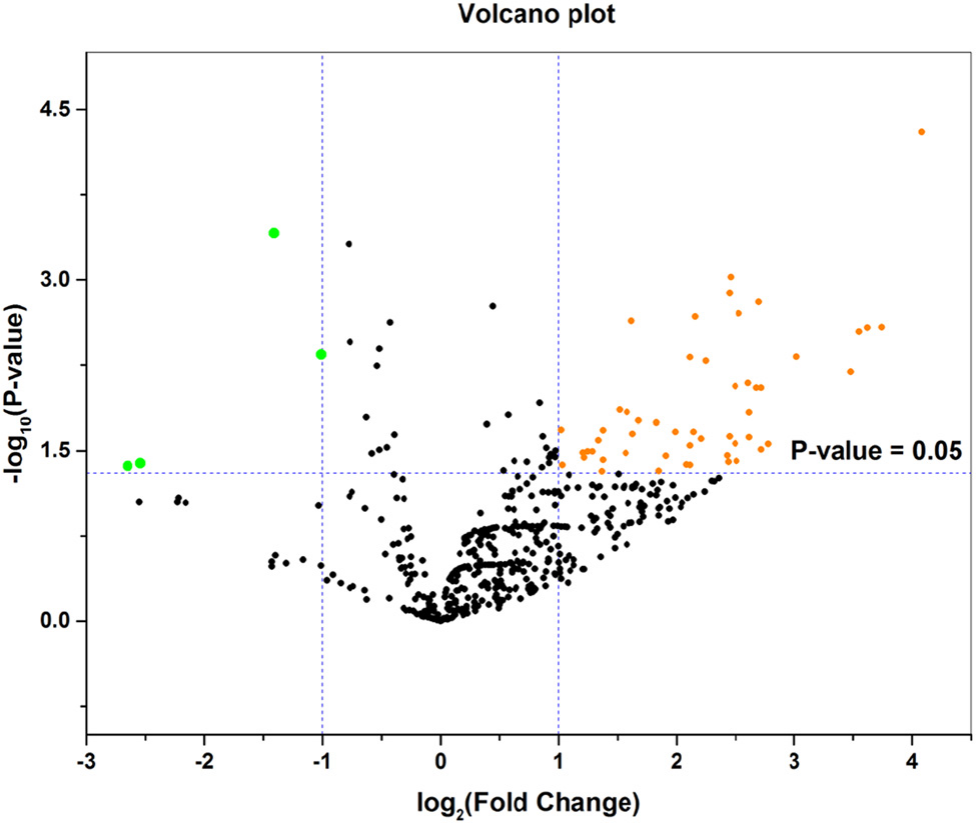
Volcano plot of the DE-miRNAs. The black points represent genes with no significant difference. The orange points represent upregulated genes screened based on the |log FC| > 1.0 and *P*-value <0.05. The green points represent downregulated genes screened based on the |log FC| > 1.0 and *P*-value <0.05.

The downloaded gene expression microarray dataset (GSE100508) included 14 healthy controls (GSM2685625–GSM2685638) and 14 melanoma patients (GSM2685639–GSM2685652). The data were normalized using the limma R package, and the miRNA expression in pEVs was compared between the two groups (Figure 1). The screening criteria were set to a *P*-value of <0.05 and |log FC| > 1, and the results showed a total of 55 DE-miRNAs (51 upregulated miRNAs and 4 downregulated miRNAs) and displayed by a heat map (Figure 2). The top ten upregulated miRNAs and all downregulated miRNAs were selected and are listed in Table 1, and volcano maps were used to show the distribution of all DE-miRNAs (Figure 3). Upregulated miRNAs were sorted according to the *P*-value, and hsa-miR-765, hsa-miR-362-3p, hsa-miR-550a-3p, hsa-miR-3907 and hsa-miR-500a-3p were the top five upregulated DE-miRNAs. hsa-miR-1238, hsa-miR-1228-3p, hsa-miR-10a-5p and hsa-miR-150-5p were the downregulated DE-miRNAs. Retrieving the miRTarBase database, a total of 3,083 candidate TGs were diagnostically correlated with the top ten upregulated DE-miRNAs, and 1,351 candidate TGs were diagnostically associated with the downregulated DE-miRNAs.

**Table 1:** Top ten upregulated and all downregulated DE-miRNAs in pEVs between melanoma and health control

ID	Adj. *P*-value	*P*-value	*T*	*B*	log FC	Regulated
hsa-miR-765	0.0272	0.0000498	4.81	1.9252	4.08	Upregulated
hsa-miR-362-3p	0.1107	0.0009439	3.71	−0.6452	2.46	Upregulated
hsa-miR-550a-3p	0.1107	0.00131	3.58	−0.9311	2.45	Upregulated
hsa-miR-3907	0.1107	0.0015673	3.51	−1.0873	2.7	Upregulated
hsa-miR-500a-3p	0.1107	0.0019777	3.42	−1.2896	2.53	Upregulated
hsa-miR-199b-5p	0.1107	0.0021114	3.4	−1.3465	2.16	Upregulated
hsa-miR-15b-3p	0.1107	0.0022978	3.36	−1.42	1.62	Upregulated
hsa-miR-34a-5p	0.1107	0.0026186	3.31	−1.5335	3.74	Upregulated
hsa-miR-4284	0.1107	0.0026311	3.31	−1.5376	3.62	Upregulated
hsa-miR-215	0.1118	0.0028611	3.28	−1.6103	3.55	Upregulated
hsa-miR-1238	0.3117	0.0433103	−2.12	−3.9149	−2.65	Downregulated
hsa-miR-1228-3p	0.3087	0.0408028	−2.15	−3.8662	−2.54	Downregulated
hsa-miR-10a-5p	0.1384	0.0045602	−3.09	−2.0136	−1.01	Downregulated
hsa-miR-150-5p	0.0881	0.0003886	−4.04	0.1301	−1.41	Downregulated

### 3.2 GO annotation and KEGG pathway enrichment analysis of TGs

**Figure 4:**
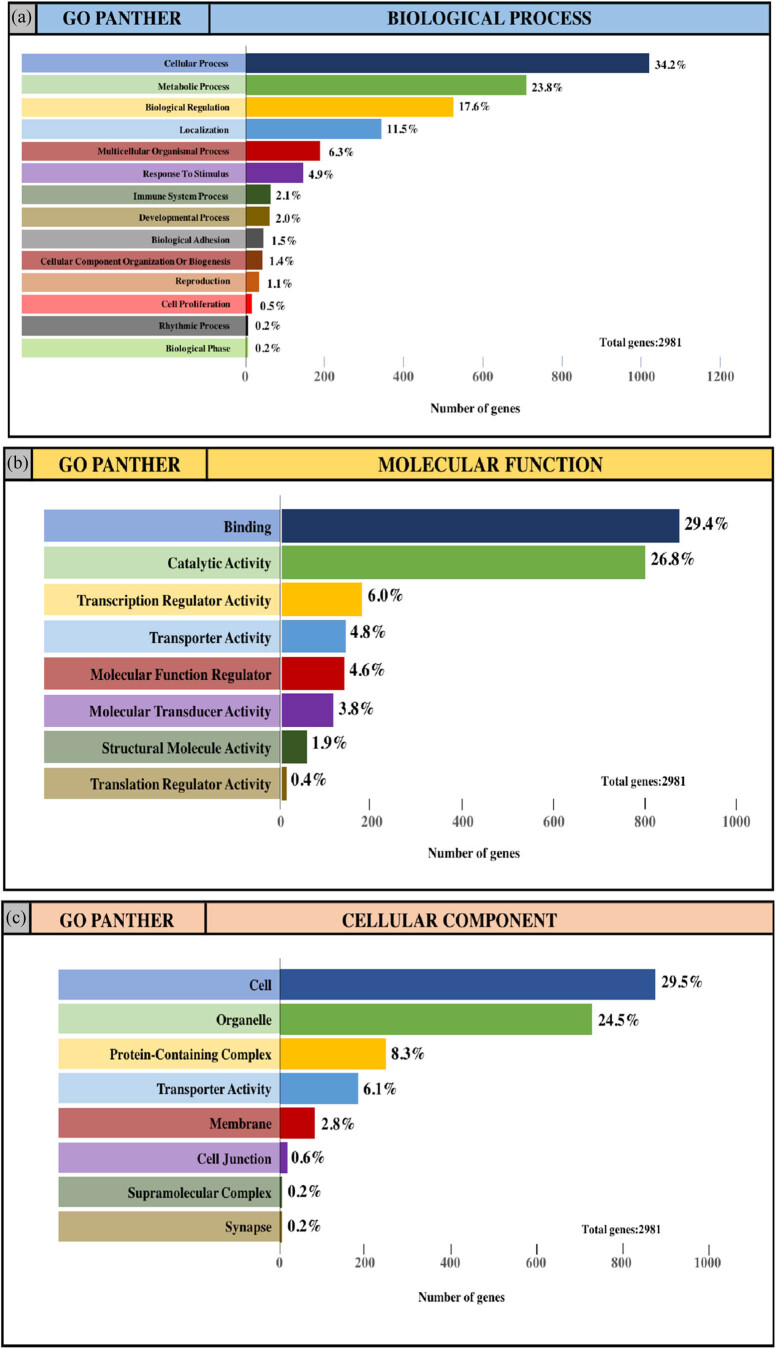

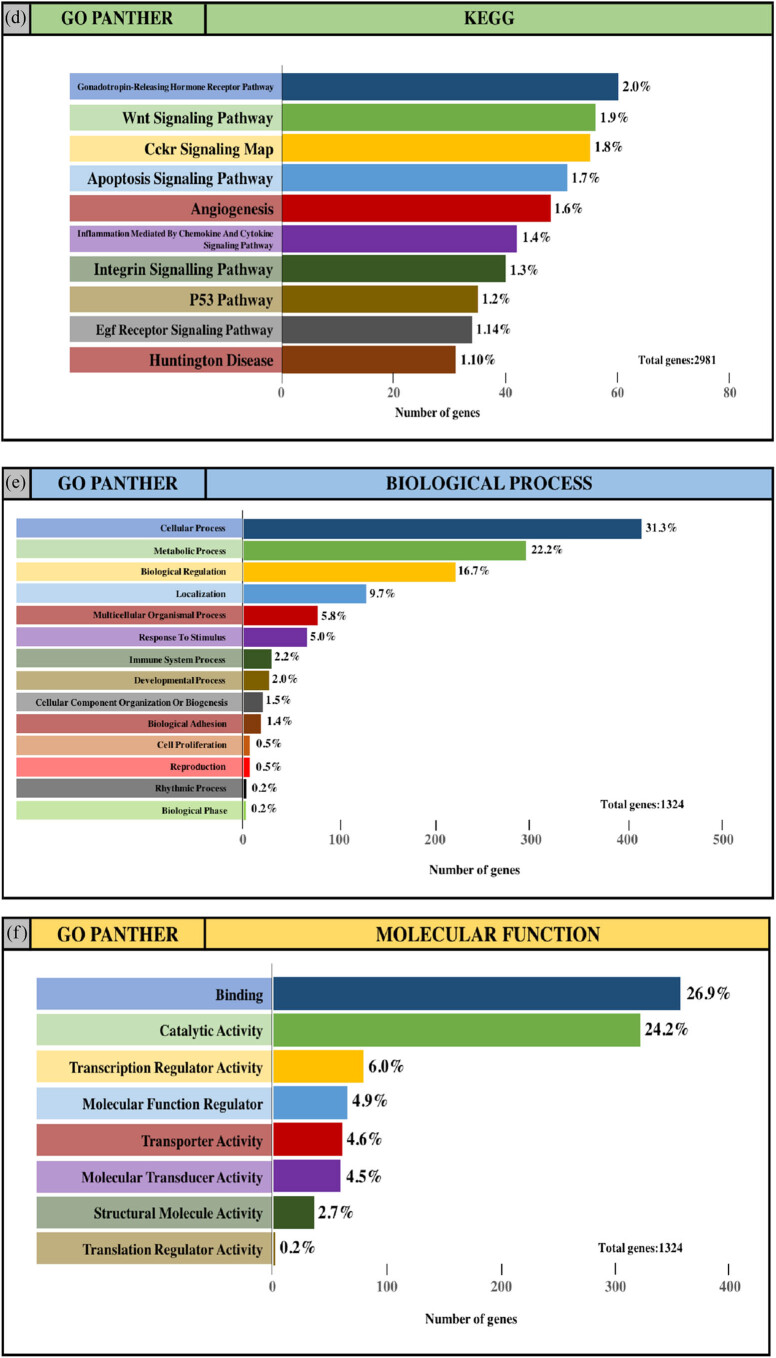

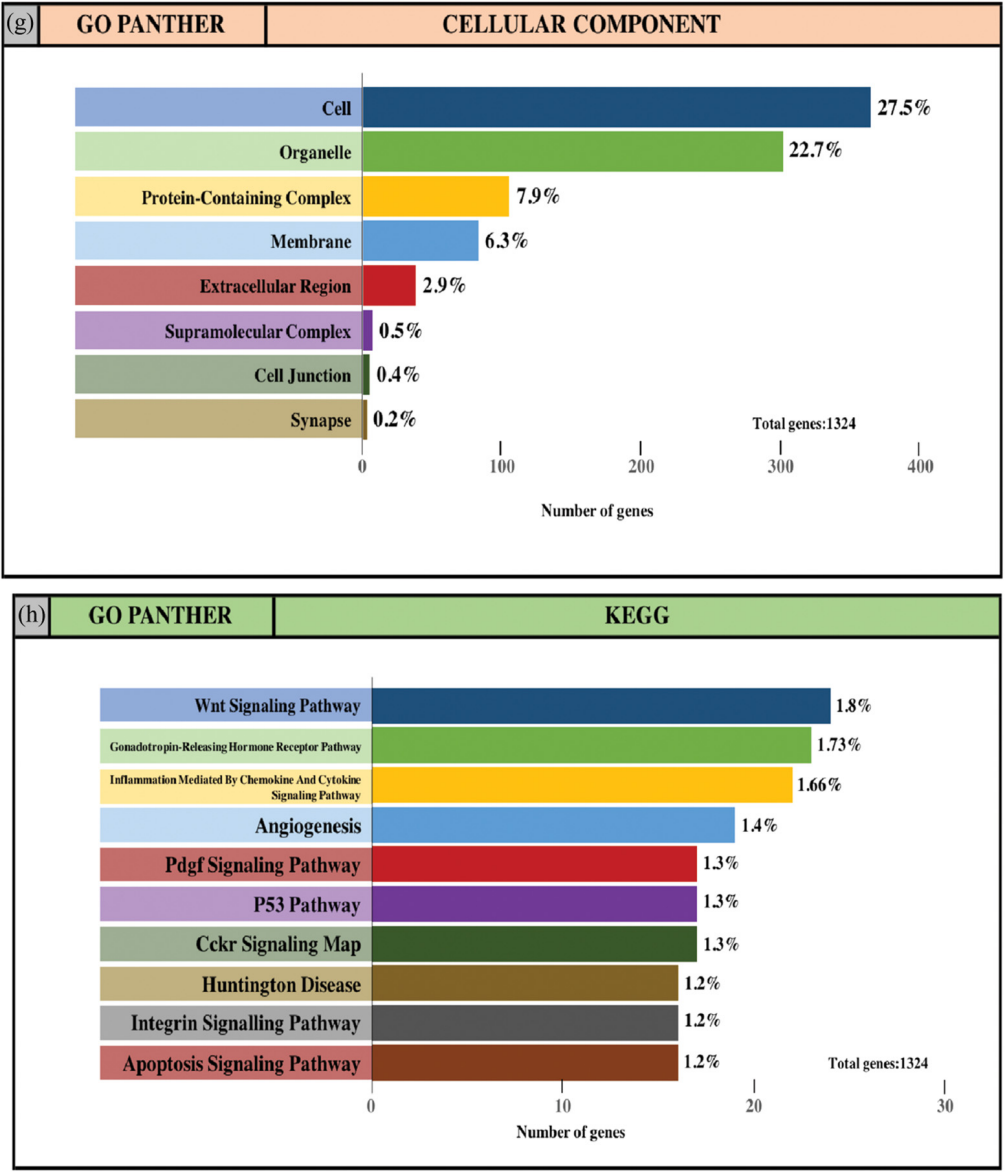
GO and pathway enrichment analysis of the predicted TG of DE-miRNAs through the PANTHER database. (a and e) GO PANTHER’s analysis on the category of “biological process” of the predicted TG of upregulated miRNAs and downregulated miRNAs, respectively. (b and f) GO PANTHER’s analysis on the category of “molecular function” of the predicted TG of upregulated miRNAs and downregulated miRNAs, respectively. (c and g) GO PANTHER’s analysis on the category of “cellular component” of the predicted TG of upregulated miRNAs and downregulated miRNAs, respectively. (d and h) GO PANTHER’s analysis on the category of “KEGG” of the predicted TG of upregulated miRNAs and downregulated miRNAs, respectively.

The integrated TGs were analyzed using the PANTHER classification system for GO annotation and KEGG pathway analysis to further reveal the enrichment of TGs in MF, BP, CC and pathways. For the BP (Figure 4a and e), most of the TGs were involved in the regulation of cellular process (34.2% and 31.3%, upregulated miRNAs and downregulated miRNAs, respectively), metabolic process (23.8% and 22.2%, upregulated miRNAs and downregulated miRNAs, respectively) and biological regulation (17.6% and 16.7%, upregulated miRNAs and downregulated miRNAs, respectively). For MF (Figure 4b and f), TGs were mainly involved in binding (29.4% and 26.9%, upregulated miRNAs and downregulated miRNAs, respectively) and catalytic activity (26.8% and 24.2%, upregulated miRNAs and downregulated miRNAs, respectively). With regard to the cellular component (Figure 4c and g), the majority of the TGs were components of the cell (29.5% and 27.5%, upregulated miRNAs and downregulated miRNAs, respectively) and organelles (24.5% and 22.7%, upregulated miRNAs and downregulated miRNAs, respectively). Subsequently, the KEGG pathway enrichment analysis results showed that the TGs were most significantly enriched in the gonadotropin-releasing hormone receptor pathway and the Wnt signaling pathway (Figure 4d and h).

### 3.3 Analysis of the PPI network and the miRNA target network

**Figure 5:**
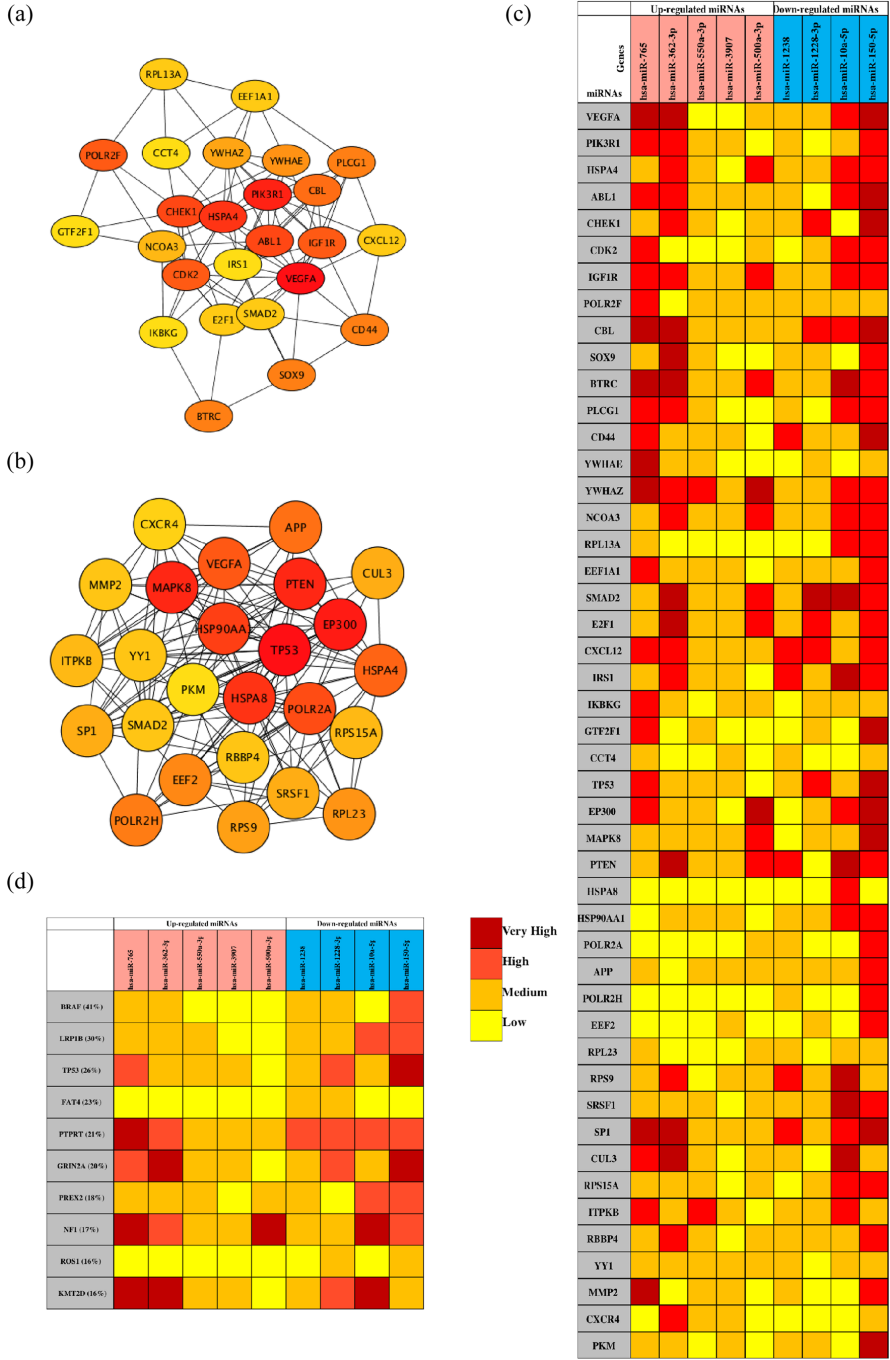

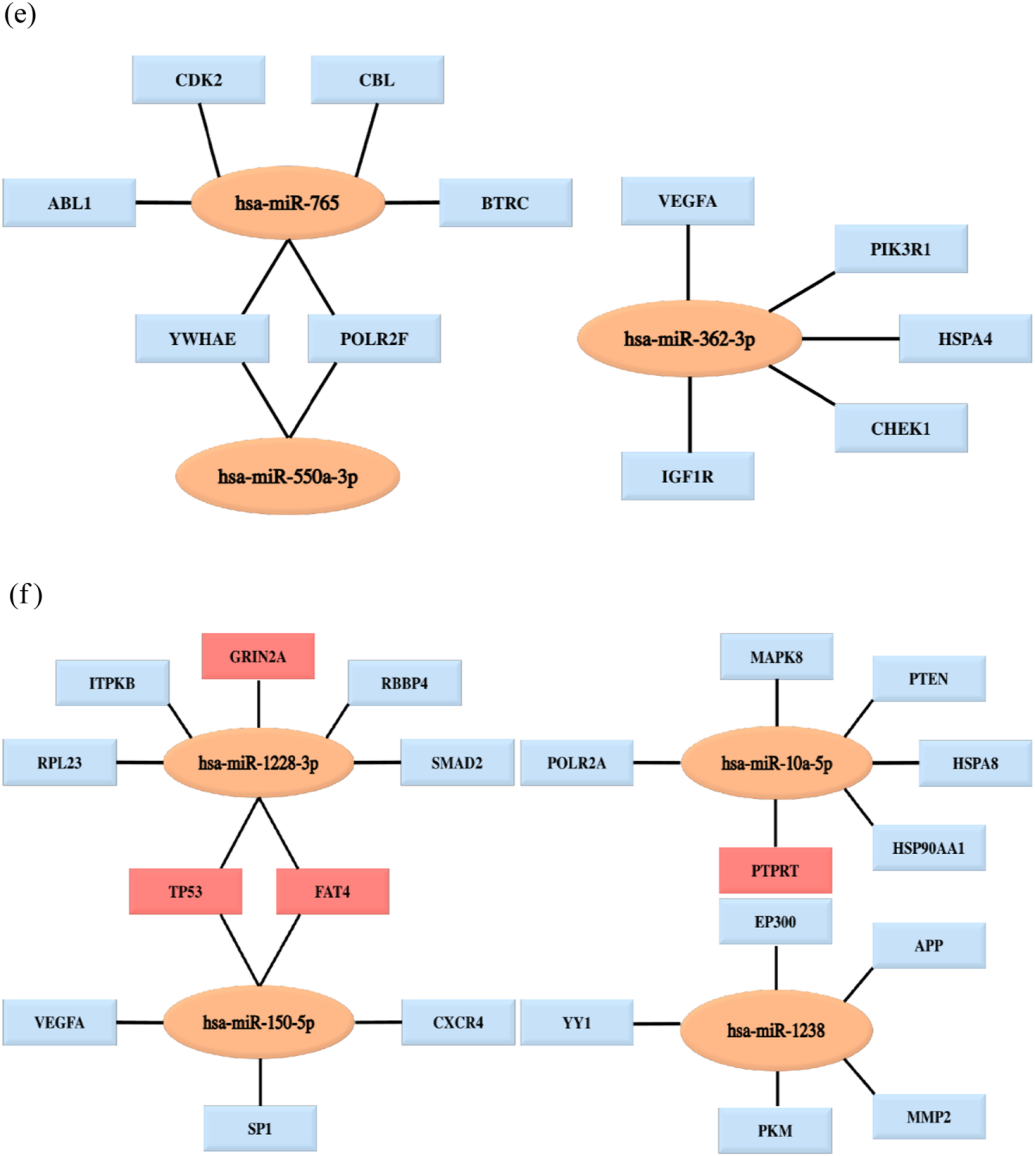
Analysis of the PPI network and the miRNA target network. (a) The mapped networks for the top 25 hub genes for upregulated miRNAs. (b) The mapped networks for the top 25 hub genes for downregulated miRNAs. (c) The mirDIP analysis of interaction levels between hub genes and DE-miRNAs. (d) The mirDIP analysis of interaction levels between the main mutated genes and DE-miRNAs. (e) The mapped networks for upregulated miRNAs and hub genes. (f) The mapped networks for downregulated miRNAs and hub genes. The red gene indicates that it is both a mutant gene and a core gene.

The PPI network analysis is important for understanding the mechanisms of biosignal and energy metabolism and the functional linkages between proteins under specific physiological conditions of disease. In this study, we used the STRING database to perform PPI network analysis on the predicted TGs of the top five most upregulated and all downregulated DE-miRNAs. Then, the hub genes with the highest degree of the first 25 nodes were obtained by the Degree algorithm (Table 2) and visualized with Cytoscape software (Figure 5a and b). Regarding the upregulated miRNAs, VEGFA, PIK3R1, HSPA4, ABL1 and CHEK1 were the top five hub genes, while TP53, EP300, MAPK8, PTEN and HSPA8 were the top five hub genes for the downregulated miRNAs.

**Table 2:** Hub genes in the PPI networks

Upregulated miRNAs		Downregulated miRNAs	
Gene	Degree	Gene	Degree
VEGFA	64	TP53	156
PIK3R1	42	EP300	85
HSPA4	39	MAPK8	80
ABL1	31	PTEN	80
CHEK1	31	HSPA8	78
CDK2	30	HSP90AA1	77
IGF1R	30	POLR2A	70
POLR2F	30	VEGFA	67
CBL	29	HSPA4	65
SOX9	28	APP	64
BTRC	28	POLR2H	50
PLCG1	28	EEF2	48
CD44	28	RPL23	47
YWHAE	26	RPS9	46
YWHAZ	25	SRSF1	45
NCOA3	24	SP1	45
RPL13A	22	CUL3	45
EEF1A1	22	RPS15A	42
SMAD2	22	ITPKB	42
E2F1	22	RBBP4	41
CXCL12	22	YY1	41
IRS1	21	SMAD2	41
IKBKG	21	MMP2	41
GTF2F1	21	CXCR4	40
CCT4	21	PKM	39

Ten mutated genes most related to melanoma were retrieved from the COSMIC database. The mutated genes were BRAF (41%), LRP1B (30%), TP53 (26%), FAT4 (23%), PTPRT (21%), GRIN2A (20%), PREX2 (18%), NF1 (17%), ROS1 (16%) and KMT2D (16%). Then, the mirDIP online database was used to analyze the interaction levels of DE-miRNA-mutant genes and DE-miRNA-hub genes, and the associated heat maps were used to display the interaction levels (Figure 5c and d). Regarding the interaction levels of DE-miRNAs-hub genes, the upregulated hsa-miR-765, hsa-miR-362-3p and hsa-miR-500a-3p and the downregulated hsa-miR-10a-5p and hsa-miR-150-5p were most relevant to hub genes. Regarding the interaction levels of DE-miRNA-mutant genes, the upregulated hsa-miR-765 and hsa-miR-362-3p and the downregulated hsa-miR-10a-5p, hsa-miR-150-5p and hsa-miR-122-3p were determined to be strongly correlated with mutant genes. Subsequently, we compared the TGs of the aforementioned DE-miRNAs with the hub genes and the mutated genes. The top five genes with the highest degree were selected and plotted on the DE-miRNA-gene association map (Figure 5e and f). The results showed that the upregulated hsa-miR-765 and hsa-miR-500a-3p could jointly regulate the YWHAE and POLAR2F genes, and the downregulated hsa-miR-1228-3p and hsa-miR-150-5p could jointly regulate the TP53 and FAT4 genes. In addition, notably, the mutant genes did not overlap with the hub genes of the upregulated miRNAs, while the TP53 and FAT4 genes were both hub genes and mutant genes for the downregulated hsa-miR-1228-3p and hsa-miR-150-5p.

### 3.4 Prognostic analysis

**Figure 6:**
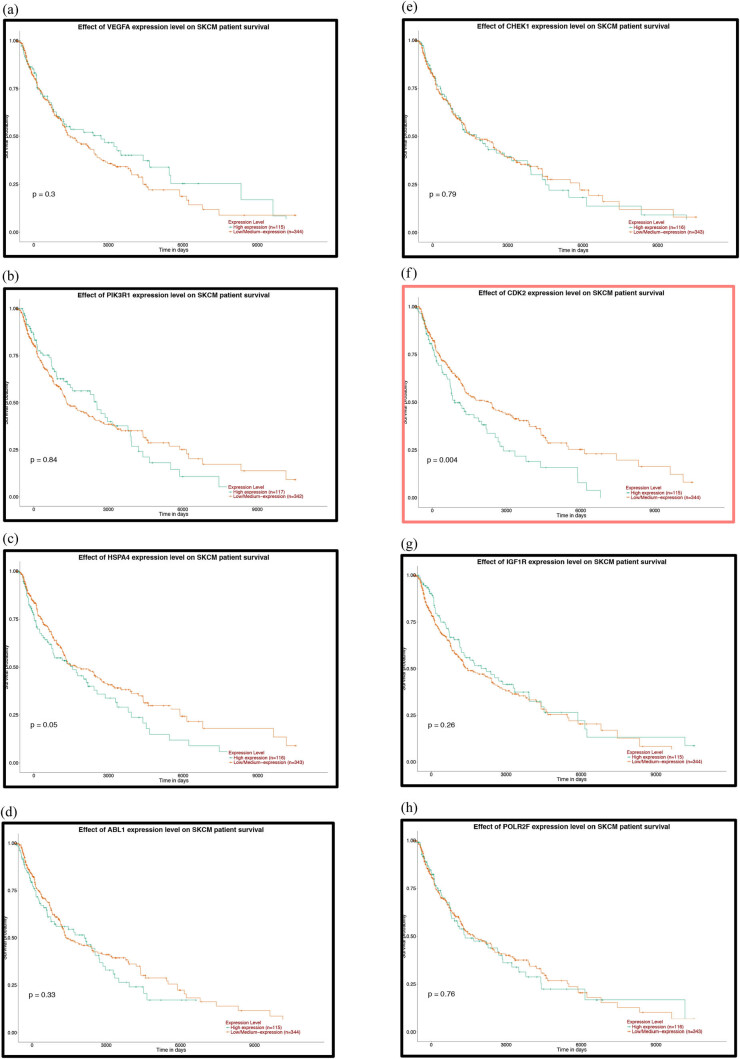

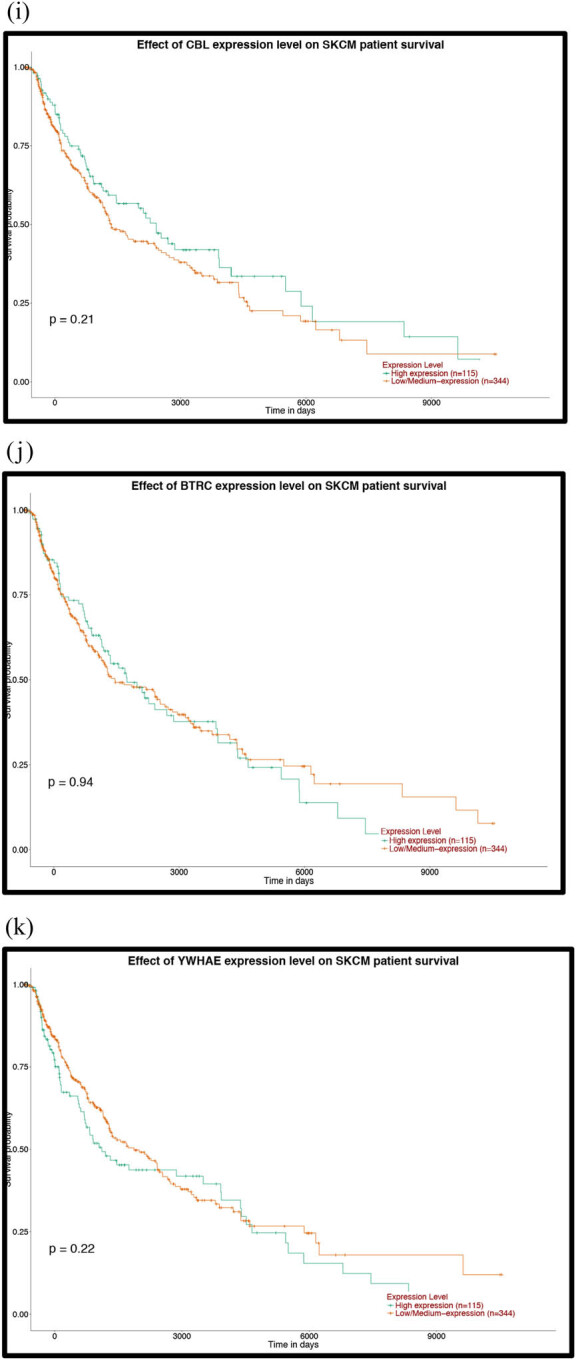
Prognostic analysis of the mutant genes and the hub genes of the downregulated miRNAs with the UALCAN database: (a) TP53, (b) FAT4, (c) PTPRT, (d) GRIN2A, (e) EP300, (f) MAPK8, (g) PTEN, (h) HSPA8, (i) HSP90AA1, (j) APP, (k) POLR2A, (l) RPL23, (m) SP1, (n) ITPKB, (o) RBBP4, (p) YY1, (q) SMAD2, (r) MMP2 and (s) CXCR4. *P* < 0.05 was considered statistically significant.

**Figure 7:**
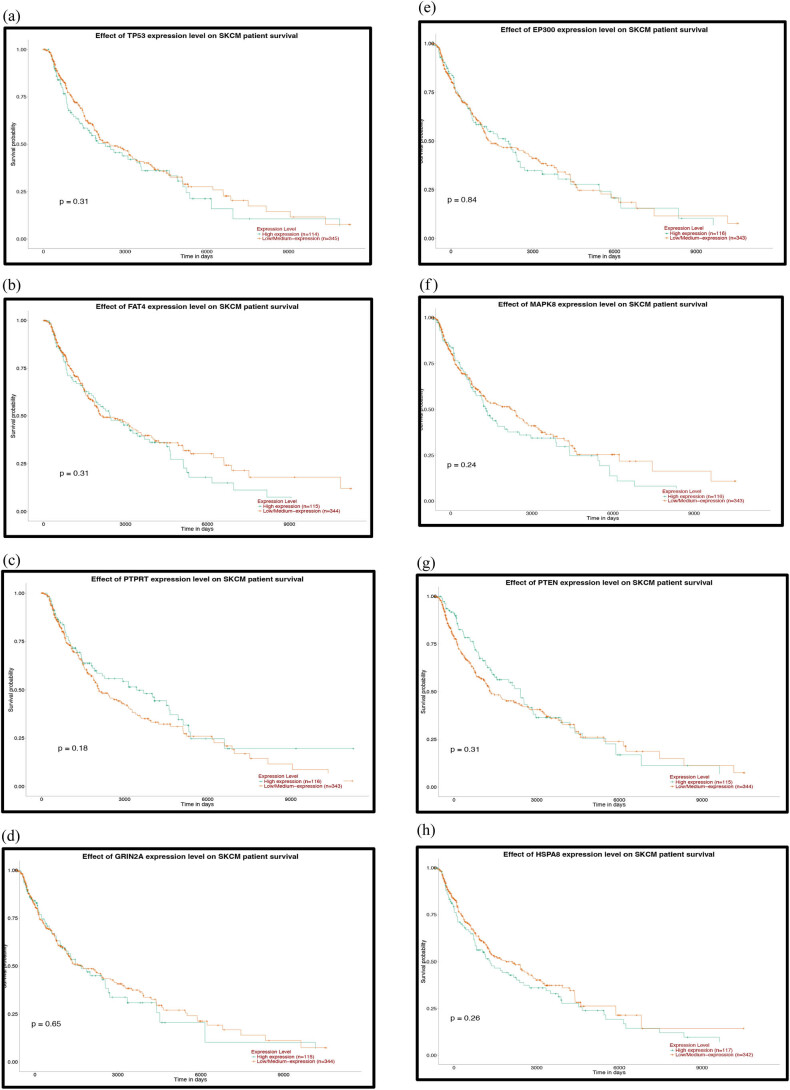

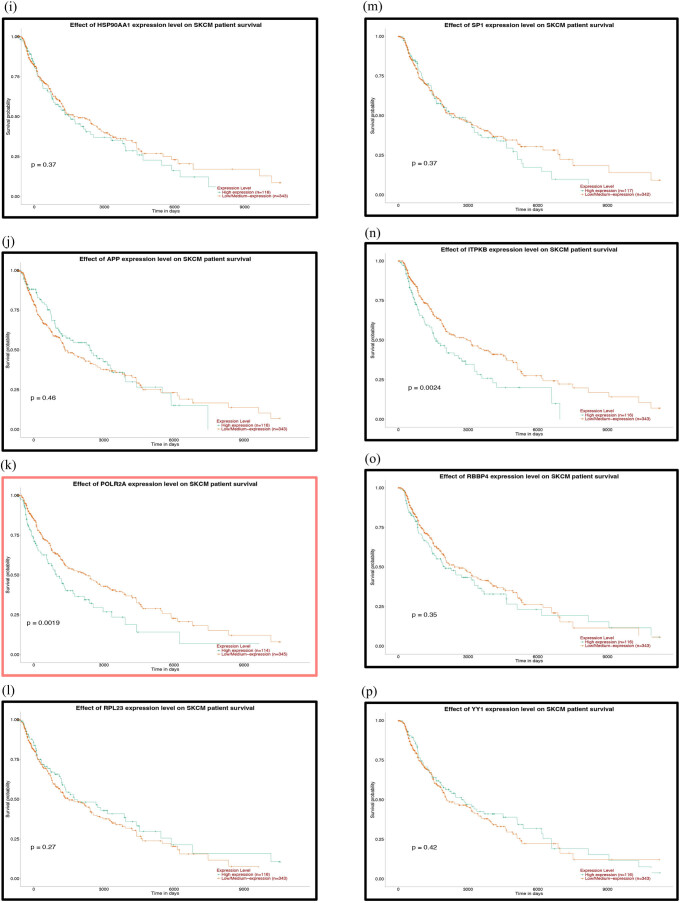

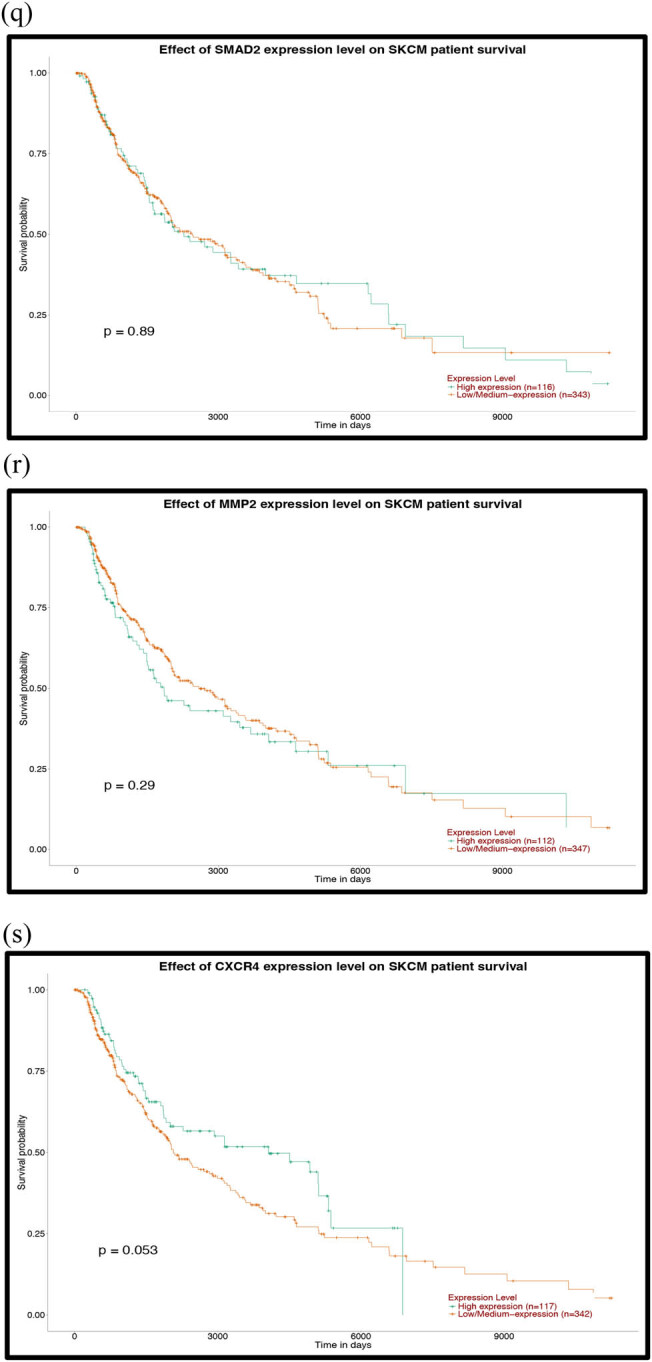
Prognostic analysis of the hub genes of the upregulated miRNAs with the UALCAN database: (a) VEGFA, (b) PIK3R1, (c) HSPA4, (d) ABL1, (e) CHEK1, (f) CDK2, (g) IGF1R, (h) POLR2F, (i) CBL, (j) BTRC and (k) YWHAE. *P* < 0.05 was considered statistically significant.

**Figure 8:**
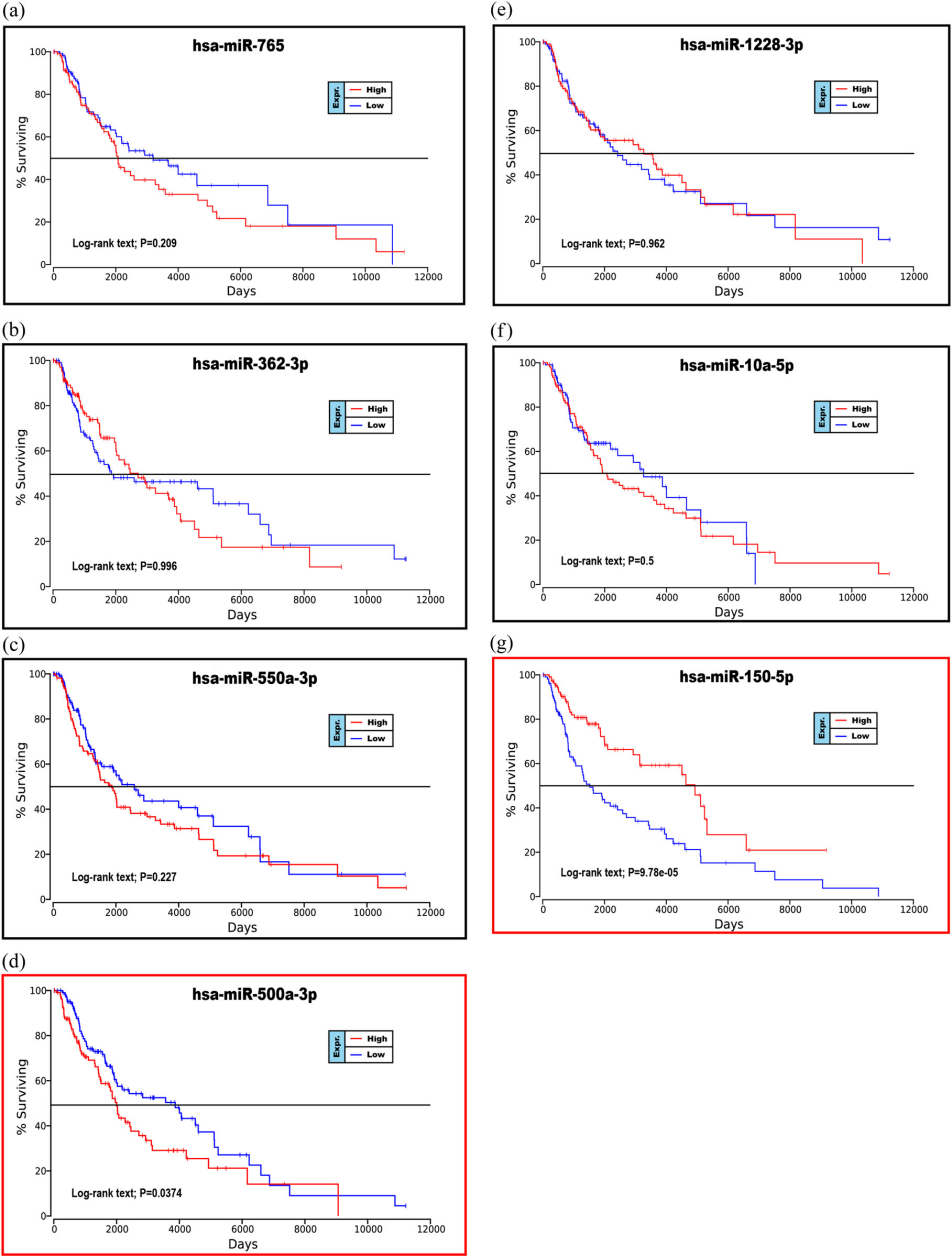
Prognostic analysis of the upregulated or downregulated DE-miRNAs with the OncoLnc database: (a) hsa-miR-765, (b) hsa-miR-362-3p, (c) hsa-miR-550a-3p, (d) hsa-miR-500a-3p, (e) hsa-miR-1228-3p, (f) hsa-miR-10a-5p and (g) hsa-miR-150-5p. *P* < 0.05 was considered statistically significant.

To further explore the relationship between patient prognosis and molecular markers, including the DE-miRNAs, the hub genes and the mutant genes. The UALCAN and OncoLnc online websites were used to analyze the relationship between these genes and the overall survival (OS) rate of patients with melanoma. We selected the top five genes with the highest degree of the above DE-miRNAs (the upregulated hsa-miR-765, hsa-miR-362-3p and hsa-miR-500a-3p and the downregulated hsa-miR-10a-5p and hsa-miR-150-5p) and the mutant genes associated with the downregulated genes for OS analysis. The results showed that there was no correlation between the mutated genes and the OS rate of patients (Figure 6a–d). High expression of CDK2 and POLR2A genes in melanoma patients significantly reduced OS (Figures 7a–k and 7e–s). The results of the correlation between the top five DE-miRNAs and the OS rate showed that there were no data for hsa-mir-3907 and hsa-mir-1238, and the expression of hsa-mir-765, hsa-mir-362-3p and hsa-mir-550a-3p had no correlation with the OS rate (Figure 8a–c, e and f). However, the OS rate of patients with high expression of hsa-mir-500a-3p and low expression of hsa-mir-150-5p was significantly decreased (Figure 8d and g).

## 4 Discussion

Melanoma originates from the neuroectoderm, which is transformed from the malignant transformation of melanocytes and is highly invasive and metastatic. Melanoma has become the most lethal type of skin cancer. Surgical resection is still the first choice for the treatment of early melanoma [3]. Advanced melanoma requires surgery combined with chemotherapy, radiotherapy, immunotherapy and targeted therapy, but the prognosis of advanced melanoma remains unsatisfactory. Therefore, it is important to identify biomarkers that can be used for the early diagnosis and prognosis of melanoma. As an important information transfer material in the tumor microenvironment, pEVs have attracted the attention of scientists. A large number of studies have reported that DE-miRNAs in pEVs can be used as potential molecular markers for the diagnosis and treatment of lung cancer, prostate cancer and colorectal cancer [22,23,24]. Therefore, we hypothesized that DE-miRNAs in pEVs of melanoma patients may also be potential molecular targets for the diagnosis and treatment of melanoma. In this study, the GSE100508 microarray data were downloaded from the GEO database, and 55 DE-miRNAs in pEVs of melanoma patients were identified by differential analysis compared with pEVs in normal controls. In the pEVs of melanoma, hsa-miR-765 was the main upregulated miRNA, and hsa-miR-1238 was the main downregulated miRNA.

It has been shown that hsa-miR-765 and hsa-miR-1238 are associated with hepatocellular carcinoma, osteosarcoma, glioblastoma and other malignant tumors [25,26,27]. Regarding hepatocellular carcinoma, hsa-miR-765 was significantly overexpressed in hepatoma cell lines and tissues compared with normal hepatocytes and paracancerous tissues. Then, it was verified that overexpression of hsa-miR-765 promotes the proliferation and tumorigenicity of hepatoma cells [25]. Regarding osteosarcoma, miR-300 was also highly enriched in human osteosarcoma cells and plays an important role in the regulation of developmental processes, cell processes and cell signaling pathways (such as Wnt, MAPK and p53 signaling pathway) [28]. Similarly, miR-1238 was significantly enriched in the serum of glioblastoma patients compared to healthy individuals, and loss of miR-238 may lead to the formation of drug-resistant glioblastoma via the CAV1/EGFR pathway [27]. In addition, a recent study reported that miR-765 plays an important role in the development of malignant melanoma [29]. The promoter of miR-765 binds directly to HOXB9, promotes self-transcription and then targets FOXA2 to regulate FOXA2, resulting in a decrease in FOXA2 and an increase in melanoma tumor stem cells. At the same time, the miR-765-mediated FOXA pathway maintains the characteristics of cancer stem cells and contributes to the survival of melanoma.

TGs are involved in the development of melanoma. Therefore, GO annotation and KEGG pathway enrichment analysis were performed on differential genes to explore the expression changes of TGs in the BP of melanoma. All TGs were primarily enriched in the BP category in cellular and metabolic processes. Previous studies have shown that key genes that regulate cellular processes maintain a balance between the proliferation and differentiation of melanocyte stem cells [29,30,31]. The acquisition or loss of key gene functions will disrupt the balance of melanocyte stem cells, leading to the development and progression of melanoma. In addition, recent studies have shown that there is heterogeneity in the metabolism of melanoma, which can utilize various resources to promote tumor development. At the same time, the metabolic process of melanoma has an important impact on the aggressiveness of the tumor [32]. The TGs of upregulated miRNAs were mainly enriched in the gonadotropin-releasing hormone receptor pathway, while the TGs that downregulated miRNAs were primarily enriched in the Wnt signaling pathway. Previous studies have shown that the gonadotropin-releasing hormone receptor pathway is not only a cancer cell autocrine regulatory factor but is also expressed in hormone-independent melanoma, which can negatively regulate the proliferation and metastasis of melanoma cells [33,34,35]. The Wnt signaling pathway controls key steps in cell polarity, proliferation and migration [36]. Kulikova et al. [37] found that abnormal activation of the Wnt pathway is one of the key signals leading to the development of melanoma. The typical and atypical Wnt pathways are involved in the formation and metastasis of melanoma, respectively.

Hub genes may play an important role in the onset and progression of melanoma. Through analysis of the PPI network of TGs, it was found that VEGFA is the main hub gene for upregulated miRNAs, while TP53 is the main hub gene for downregulated miRNAs. VEGF, a vascular endothelial growth factor, is involved in the formation of blood vessels in the tumor and the expansion of the vascular network. Erhard et al. [38] found that the invasive potential of metastatic melanoma is associated with vascularization. Therefore, VEGF may play an important role in the development of melanoma. A number of studies have shown that VEGF levels in plasma and lymph nodes of melanoma patients are significantly higher than those in the control group, which may contribute to the diagnosis of melanoma micrometastasis [39,40]. TP53 is one of the most common mutations in human cancer and can lead to the rapid deterioration of tumors [41]. We found that TP53 is also one of the common mutations in skin malignant melanoma by searching the COSMIC database. Regad [30] found that the encoded protein ARF is a stabilizer for p53, and patients with melanoma often exhibit p53 mutations due to frequent loss of ARF, which ultimately leads to excessive proliferation of melanoma cells. Interestingly, Kim et al. [42]. found that TP53 gene mutation resulted in high expression of p53 protein, which improved the survival rate of patients with stage III and IV melanoma. The results of this study are in contrast to the poor clinical prognosis of malignant tumors with common TP53 mutations, but the specific mechanisms have not been elucidated to date.

Prognostic correlation analysis of cutaneous melanoma with molecular markers found that high expression of hsa-miR-500a-3p, CDK2 and POLR2A genes or low expression of hsa-miR-150-5p can significantly reduce OS in melanoma patients. Azimi et al. [43] found that CDK2 is a driving factor for BRAF and Hsp90 inhibitor resistance. High expression of CDK2 causes melanoma patients to rapidly develop resistance to drug-targeted therapeutic agents, which leads to a decrease in OS. Frischknecht et al. [44] found that degradation of POLAR2 promotes apoptosis in melanoma cells. Therefore, the proliferation rate of melanoma cells in patients with high POLAR2 expression is significantly higher than that in patients with low expression, resulting in low OS.

However, this study still has many limitations. Tumor EVs are currently receiving increasing attention from researchers, but relevant data sets are still scarce. The sample size of patients with melanoma pEVs needs to be expanded to better support the aforementioned findings. In addition, this study found a large number of melanoma-related DE-miRNAs and TGs that require extensive follow-up research to validate.

## 5 Conclusions

This study analyzed the DE-miRNA expression in pEVs between melanoma patients and normal controls by bioinformatics. Then, the TGs associated with melanoma diagnosis and prognosis were predicted and analyzed. These DE-miRNAs and TGs may become new molecular targets for the early diagnosis and treatment of malignant melanoma.
